# Differential Zoledronate Adsorption by Commercially Available Biphasic Calcium Phosphate Bone Substitutes

**DOI:** 10.3390/ph19081281

**Published:** 2026-08-13

**Authors:** Siri Paulo, Mafalda Laranjo, Ana Margarida Abrantes, Ricardo Jorge Teixo, Diogo A. Fonseca, João Mendes-Abreu, José Pedro Figueiredo, Maria Filomena Botelho, Manuel Marques Ferreira, Arménio Serra

**Affiliations:** 1 Institute of Endodontics, Faculty of Medicine, University of Coimbra, 3000-548 Coimbra, Portugal; mmferreira@fmed.uc.pt; 2 Center for Innovative Biomedicine and Biotechnology (CIBB), University of Coimbra, 3000-548 Coimbra, Portugal; mafaldalaranjo@fmed.uc.pt (M.L.); ricardo.teixo@fmed.uc.pt (R.J.T.); mfbotelho@fmed.uc.pt (M.F.B.); 3 Area of Environment, Genetics and Oncobiology (CIMAGO), Faculty of Medicine, Coimbra Institute for Clinical and Biomedical Research (iCBR), University of Coimbra, 3000-548 Coimbra, Portugal; 4 Clinical Academic Center of Coimbra (CACC), University of Coimbra, 3000-354 Coimbra, Portugal; 5 Institute of Biophysics, Faculty of Medicine, University of Coimbra, 3000-548 Coimbra, Portugal; 6 Faculty of Pharmacy, University of Coimbra, 3000-548 Coimbra, Portugal; diogo.fonseca@ff.uc.pt; 7 Centro Hospitalar e Universitário de Coimbra, 3004-561 Coimbra, Portugal; jruideabreu@ulscoimbra.minsaude.pt (J.M.-A.); 7876@ulscoimbra.minsaude.pt (J.P.F.); 8 Laboratory for Evidence-Based Sciences and Precision Dentistry, University Coimbra, 3000-548 Coimbra, Portugal; 9 Centre for Mechanical Engineering, Materials and Processes-CEMMPRE, Chemical Engineering Department, University of Coimbra, 3000-548 Coimbra, Portugal; 10 Department of Chemical Engineering, Faculty of Sciences and Technology, University of Coimbra, 3030-790 Coimbra, Portugal; aserra@eq.uc.pt

**Keywords:** bisphosphonates, osteonecrosis of jaw, calcium phosphates, synthetic bone

## Abstract

**Background/Objectives**: Osteonecrosis of the jaw, a non-healing wound, is a side effect in patients treated with nitrogen-containing bisphosphonates (e.g., zoledronate) after some oral procedures. Considering zoledronate toxicity in surgical wounds and the capacity of calcium phosphate synthetic compounds to adsorb it, the aim of this work was to demonstrate and elucidate the mechanism underlying this adsorption. **Methods**: Four different calcium phosphate synthetic bone substitutes were used in this study: AdBone^®^ BCP (0.5–1 mm), Guidor^®^ easy-graft (0.5–1 mm), Maxresorb^®^ (0.5–1 mm) and Maxresorb^®^ (0.8–1.5 mm). The materials were incubated in aqueous zoledronate solutions. Samples of the solutions obtained after 1, 2, 3, 6, 24, 48, 72 and 120 h of conditioning were then analyzed by spectrophotometry to measure their absorbance and subsequently characterized by micro-elemental analysis. **Results**: All four materials demonstrated time-dependent zoledronate adsorption. AdBone^®^ BCP achieved the most rapid and sustained sequestration, reaching 80% reduction at 24 h and stabilizing at 85% from 72 h onwards without re-release. Maxresorb^®^ formulations showed comparable kinetics through 72 h (70–75%) but exhibited partial re-release at 120 h. Guidor^®^ easy-graft showed negligible adsorption through the first 24 h, consistent with the physical barrier effect of its PLGA coating. Spectrophotometric findings were independently confirmed by elemental analysis. **Conclusions**: This work confirms zoledronate adsorption by calcium phosphate synthetic biomaterials. Among the tested materials, AdBone^®^ BCP demonstrated the most favorable adsorption profile, with kinetics well matched to the critical window of alveolar wound healing. These findings support the use of biphasic calcium phosphate bone substitutes as a local strategy to reduce free zoledronate bioavailability at the surgical site, potentially lowering the risk of MRONJ in patients undergoing dentoalveolar procedures under bisphosphonate therapy.

## 1. Introduction

Bisphosphonates are medications for reducing osteoclast-mediated bone resorption in clinical conditions such as osteoporosis and for modulating the progression of bone metastases in malignant tumors [1,2]. Zoledronate (ZOL) is one of the most widely prescribed BPs in oncology settings to manage bone metastases of breast, prostate and lung cancer, multiple myeloma and malignancy-related skeletal complications [3]. The activation of bone remodeling favors the release of growth factors from the bone matrix and the proliferation of metastases [1,2]. Additionally, it reduces bone fractures and pain and works as a supportive treatment [4]. ZOL is a 3rd-generation nitrogenous heterocyclic bisphosphonate remaining in the bone for at least 6 years after administration is stopped [5,6,7]. Bisphosphonates are the predominant drugs underlying medication-related osteonecrosis of the jaw (MRONJ). Current clinical guidelines recommend that oncologists and other prescribing physicians inform patients of the risk of MRONJ prior to initiating treatment with antiresorptive or antiangiogenic agents and ensure timely referral to dental practitioners for preventive oral assessment before therapy begins [1,2].

MRONJ is characterized by an area of exposed bone necrosis in the maxillofacial region that persists for at least 8 weeks in patients with prior or current bisphosphonate (BP) treatment and no history of radiation therapy to the head and neck area. This debilitating condition is accompanied by pain, difficulty with oral hygiene and ongoing progression, severely limiting patients’ quality of life [8].

Indeed, adequate angiogenesis and mesenchymal stem cell recruitment to the injury site are essential prerequisites for successful bone and soft tissue healing, and their disruption is increasingly recognized as a key contributor to impaired healing outcomes in bone pathology [9]. This is consistent with the antiangiogenic and cytotoxic effects of bisphosphonates on oral soft tissues described above, which compromise the local healing capacity following dentoalveolar procedures.

MRONJ may occur after dental procedures, tooth extraction being the most frequent, thus highlighting the urgent need for preventive or therapeutic intervention [10].

From a preventive standpoint, the current strategy involves careful health examination and the use of minimally invasive surgical techniques, in addition to topical and systemic infection control. However, the search for new preventive approaches focused on the cellular level has increased. Several studies confirmed that the toxicity of bisphosphonates on soft tissues (gingival fibroblasts and oral keratinocytes) impairs wound healing and contributes to the development of osteonecrosis of the jaws [11,12,13,14]; in that way, there are emerging studies that investigate, with promising results, biphasic calcium phosphate synthetic bone substitutes as a preventive therapeutic to minimize zoledronate toxicity [15,16].

**Figure 1:**
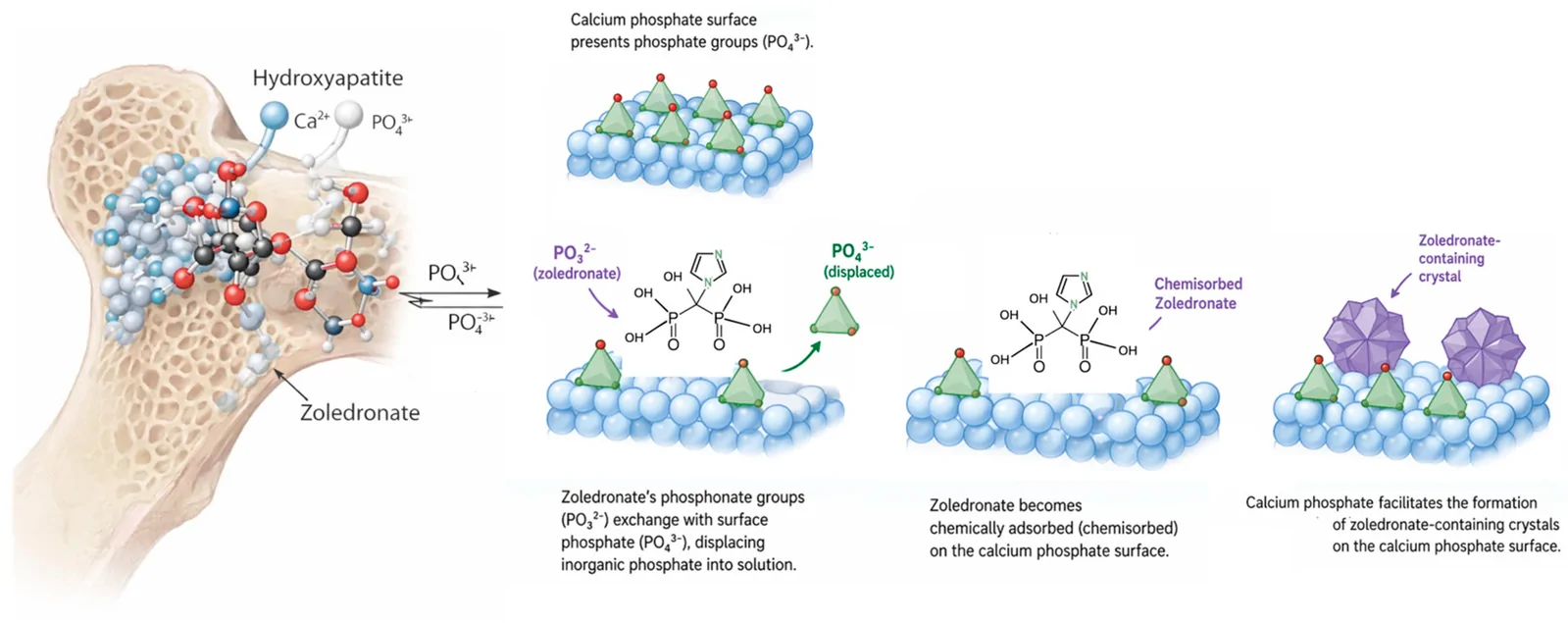
Zoledronate connected with bone hydroxyapatite. Formation of zoledronate-containing crystals on the surface of calcium phosphate with chemisorption through displacement of inorganic phosphate due to PO_3_^2−^ for PO_4_^3−^ exchange. [This figure was generated with ChatGTP (GPT-4, Open AI) according to the authors’ description].

The selection of calcium phosphate ceramics as candidate materials for zoledronate sequestration is grounded in a well-established body of evidence documenting their use as BP drug delivery systems. Calcium phosphates have long been investigated as osteotropic drug carriers precisely because of their chemical affinity for BP, whose phosphonate groups interact with ceramic surface calcium ions through the same ion-exchange and chelation mechanisms that govern bisphosphonate binding to bone mineral [17,18,19,20]. These studies consolidate this approach since they demonstrate the bonding between ZOL and calcium phosphates, specifically verifying that they promote the formation of zoledronate-containing crystals on the surface of calcium phosphate (Figure 1), leading to chemisorption of the drug through displacement of inorganic phosphate due to PO_3_^2−^ for PO_4_^3−^ exchange [21,22].

Beyond their chemical affinity, calcium phosphate ceramics offer a pharmacological safety profile validated over decades of clinical use in bone regeneration and socket preservation in dentistry, rendering them not only chemically appropriate but also clinically safe for use in patients under bisphosphonate therapy [23].

Additionally to their established safety profile, calcium phosphate-based biomaterials continue to be actively refined for bone regeneration applications, with recent developments focusing on improving their mechanical strength, injectability, and osteoinductive capacity, for instance through the incorporation of carbon nanotubes into brushite/monetite cements [24]. These ongoing advances further support the rationale for considering calcium phosphate ceramics as a versatile platform not only for structural bone repair but also for adjunctive local drug-sequestration strategies, such as the one proposed in the present study.

Building on this rationale, our group previously selected AdBone^®^ biphasic calcium phosphate (BCP), composed of 75% synthetic hydroxyapatite and 25% β-tricalcium phosphate, and demonstrated its capacity to adsorb zoledronate and protect gingival fibroblasts from ZOL-induced cytotoxicity in vitro [15], as well as to limit zoledronate toxicity and promote alveolar healing in a bisphosphonate-induced MRONJ Wistar rat extraction model in vivo [25]. These findings were subsequently corroborated by independent in vitro [16] and in vivo studies [26,27,28,29,30,31,32], reinforcing the translational potential of this therapeutic approach. Nevertheless, formal demonstration of zoledronate adsorption across the broader range of synthetic calcium phosphate bone substitutes currently available in dental practice remains lacking.

The present study therefore aimed to evaluate and compare the zoledronate adsorption capacity of four commercially available biphasic calcium phosphate ceramics sharing the same crystalline phases (Hydroxyapatite/β-Tricalcium Phosphate) but differing in their weight ratios, coating and granulometry using UV/visible spectrophotometry to quantify adsorption kinetics over time. Given that the safety and clinical efficacy of these materials as bone substitutes is already established, characterizing zoledronate adsorption behavior across different commercial formulations would support their use as a preventive strategy in dental extractions, performed in patients under bisphosphonate therapy. However, the product-specific profile should be considered when selecting the material available to the clinician.

## 2. Results

### 2.1. Spectrometric Quantification of Imidazole and of Zoledronate

**Figure 2:**
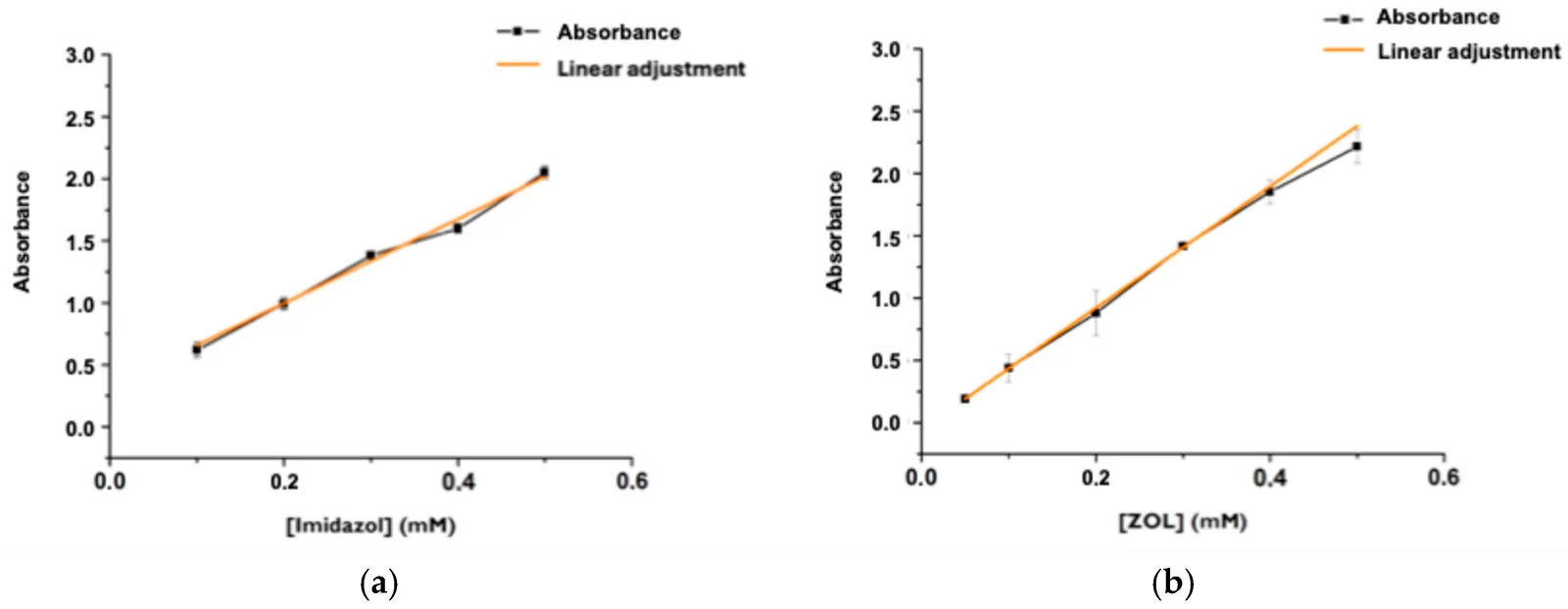
Calibration curve obtained by linear fitting between the concentration of imidazole (**a**) zoledronate (ZOL) (**b**) in millimolar and absorbance (nm).

The linear relationship between the absorbance and the concentration of imidazole, as well as the calibration curve obtained from the experimental values, is represented in Figure 2a. The same pattern was observed with the zoledronic acid solution (Figure 2b), confirming that the absorbance signal is attributable to the imidazole ring present in the zoledronate molecule. The observed linearity between concentration and absorbance of zoledronic acid enabled quantification using the calibration curve: A = 4.87·C − 0.05, where A is absorbance and C is concentration in mM. This method showed a quantification limit of 0.5 mM for the upper range of the linear response.

### 2.2. Adsorption of Zoledronic Acid to Calcium Phosphate Ceramics

**Figure 3:**
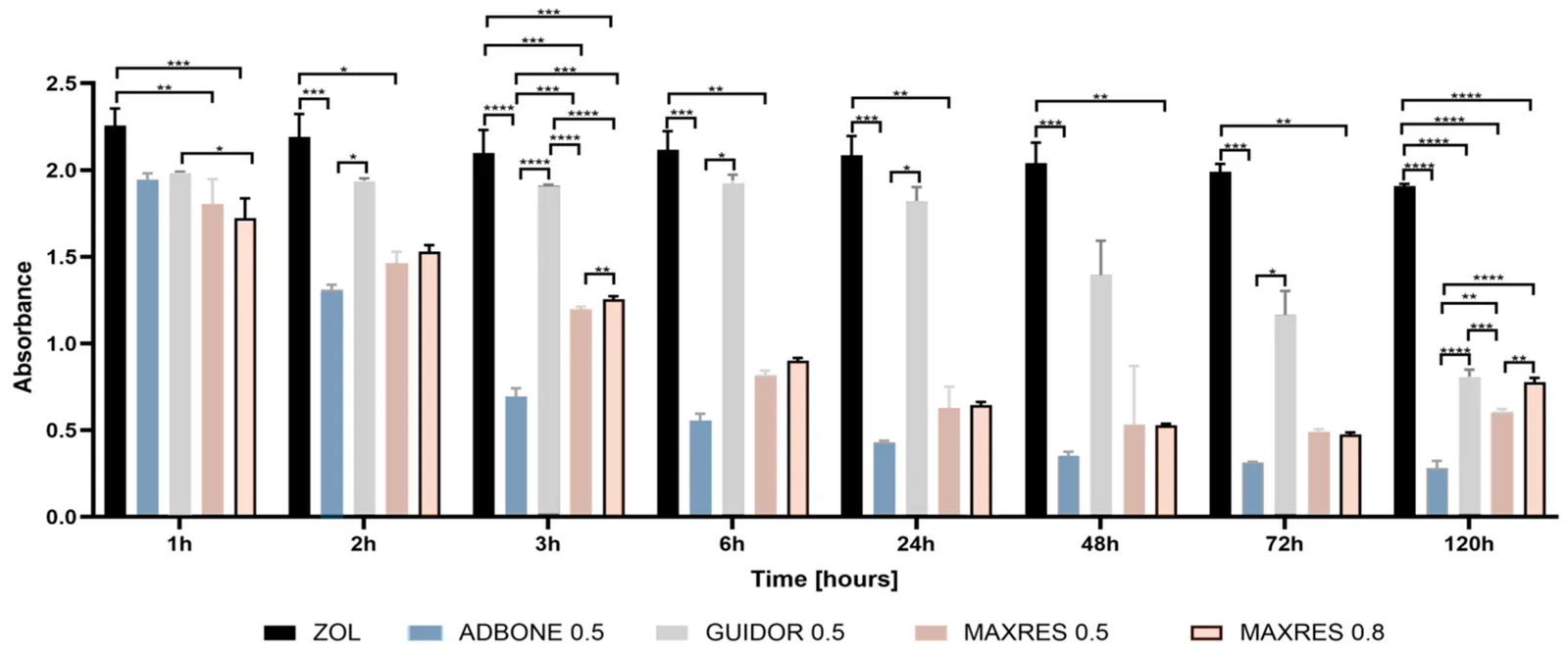
Columns represent absorbance values (at 210 nm) ± SD of 0.5 mM zoledronic acid alone (ZOL) and the association of ZOL (0.5 mM) with 0.06 g of AdBone^®^ BCP (0.5–1 mm), Guidor^®^ easy-graft (0.5–1 mm), Maxresorb^®^ (0.5–1 mm) and Maxresorb^®^ (0.8–1.5 mm) at each evaluated time point (1, 2, 3, 6, 24, 48, 72, and 120 h). Brackets indicate statistically significant differences between the connected groups at each time point. * Represents *p* < 0.05, ** *p* < 0.01, *** *p* < 0.001, **** *p* < 0.0001.

Changes in the spectrum over time indicate whether zoledronic acid was adsorbed by the biphasic calcium phosphate synthetic bone substitutes studied. The full comparative dataset across the four tested materials, as a median of six results, with the variation in absorbance of zoledronic acid (0.5 mM, pH 3.4) in the presence of 0.06 g of calcium phosphate ceramics of the commercial brands AdBone^®^ BCP (0.5–1 mm), Guidor^®^ easy-graft (0.5–1 mm), Maxresorb^®^ (0.5–1 mm) and Maxresorb^®^ (0.8–1.5 mm), is represented in Figure 3.

**Figure 4:**
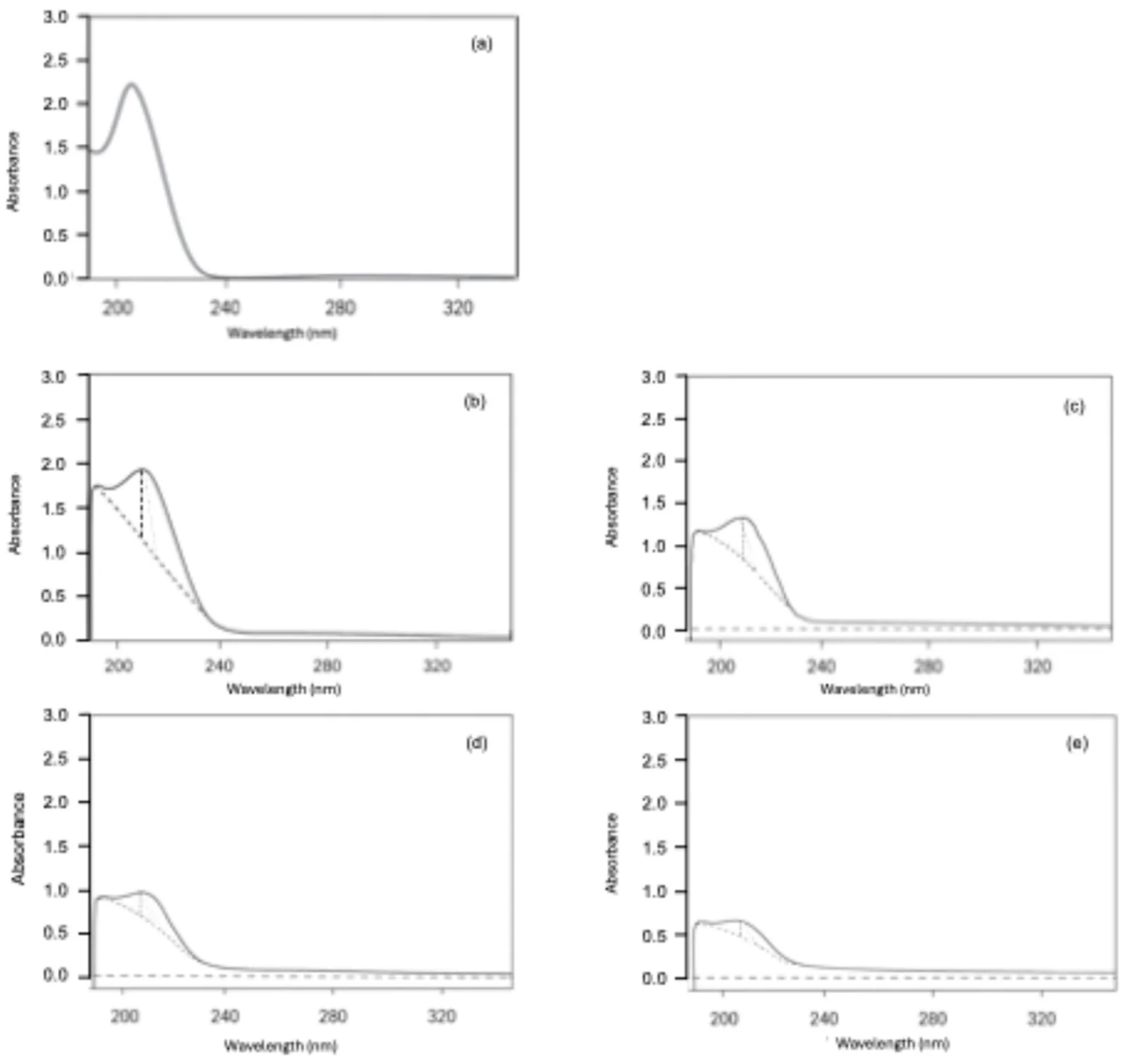
Absorption spectra representative of 0.5 mM zoledronic acid (**a**); adsorption spectra representative of curvature of association of ZOL (0.5 mM) with AdBone^®^ BCP (0.06 g) over time (**b**)—1 h; (**c**)—2 h; (**d**)—3 h and (**e**)—6 h.

To demonstrate the spectral changes underlying zoledronate quantification, a representative absorption spectrum of zoledronic acid solution (0.5 mM) alone and in the presence of AdBone^®^ BCP (0.06 g) was selected as an illustrative example due to its pronounced adsorption effect, as shown in Figure 4. The wavelength of maximum absorption was 210 nm.

The absorbance of control ZOL solutions remained constant over the 120 h evaluation period, unaffected by light exposure or temperature conditions. In contrast, all calcium phosphate materials produced a time-dependent decrease in ZOL absorbance, with significant differences between materials at multiple time points (*p* < 0.05 to *p* < 0.0001), as shown in Figure 3 and Table 1.

AdBone^®^ BCP showed the most pronounced and sustained reduction throughout the evaluation period, differing significantly from the ZOL control from 2 h onwards (2 h: *p* = 0.003 to 120 h: *p* < 0.0001), with absorbance reductions of 14% at 1 h, rising progressively to 67% at 3 h and 80% at 24 h, and reaching a plateau of 85% from 72 h through 120 h, with no evidence of re-release.

From 3 h onwards, AdBone^®^ BCP differed significantly from Guidor^®^ easy-graft at all subsequent time points (3 h: *p* < 0.0001; 6 h: *p* = 0.0159; 24 h: *p* = 0.0159; 48 h: *p* = 0.0726; 72 h: *p* = 0.0159; 120 h: *p* < 0.0001), and from both Maxresorb^®^ formulations at 3 h (MAXRES 0.5: *p* = 0.0002; MAXRES 0.8: *p* = 0.0001) and 120 h (MAXRES 0.5: *p* = 0.0028; MAXRES 0.8: *p* < 0.0001).

MaxResorb^®^ (both granulometries) differed significantly from the ZOL control from 1 h onwards (MAXRES 0.5: *p* = 0.0012; MAXRES 0.8: *p* = 0.0008), achieving reductions of 22% and 24% at 1 h and converging to 70–75% between 24 and 72 h, broadly comparable to ADBONE over this period, albeit with slightly faster initial uptake at 1 h (MAXRES 0.8 vs. ADBONE *p* = 0.5347) and a slower rate of adsorption between 1 and 3 h (MAXRES 0.5 vs. ADBONE at 3 h: *p* = 0.0002; MAXRES 0.8 vs. ADBONE at 3 h: *p* = 0.001). Both granulometries showed a partial increase in absorbance at 120 h, suggesting partial re-release of previously adsorbed ZOL, with final reductions of 70% for MAXRES 0.5 and 60% for MAXRES 0.8; the difference between granulometries at this time point was statistically significant (*p* = 0.0011).

Guidor^®^ easy-graft maintained absorbance values close to the ZOL control through the first 24 h, with reductions of only 13%, 12%, 10% and 10% at 1, 2, 3 and 6 h, respectively, differing significantly from the control only at 120 h (120 h: *p* < 0.0001). As addressed, at all time points from 3 h onwards, GUIDOR showed significantly higher absorbance than AdBone^®^ BCP (3 h: *p* < 0.001; 6 h: *p* = 0.0159; 24 h: *p* = 0.0159; 72 h: *p* = 0.0159; 120 h: *p* < 0.0001) and both Maxresorb^®^ formulations at 3 h (MAXRES 0.5: *p* < 0.0001; MAXRES 0.8: *p* < 0.0001 and MAXRES 0.5 at 120 h (*p* = 0.0003), reflecting markedly lower adsorption capacity throughout the evaluation period, with a maximum reduction of only 57% reached at 120 h.

The quantitative reduction in absorbance for each material at each time point is summarized in Table 1. These percentages were calculated from the raw absorbance values shown in Figure 3 using the zoledronic acid control solution absorbance at each corresponding time point as the reference (100%); Figure 3 and Table 1 report the same underlying dataset, expressed as absolute absorbance values and as percentage reduction, respectively.

**Figure 5:**
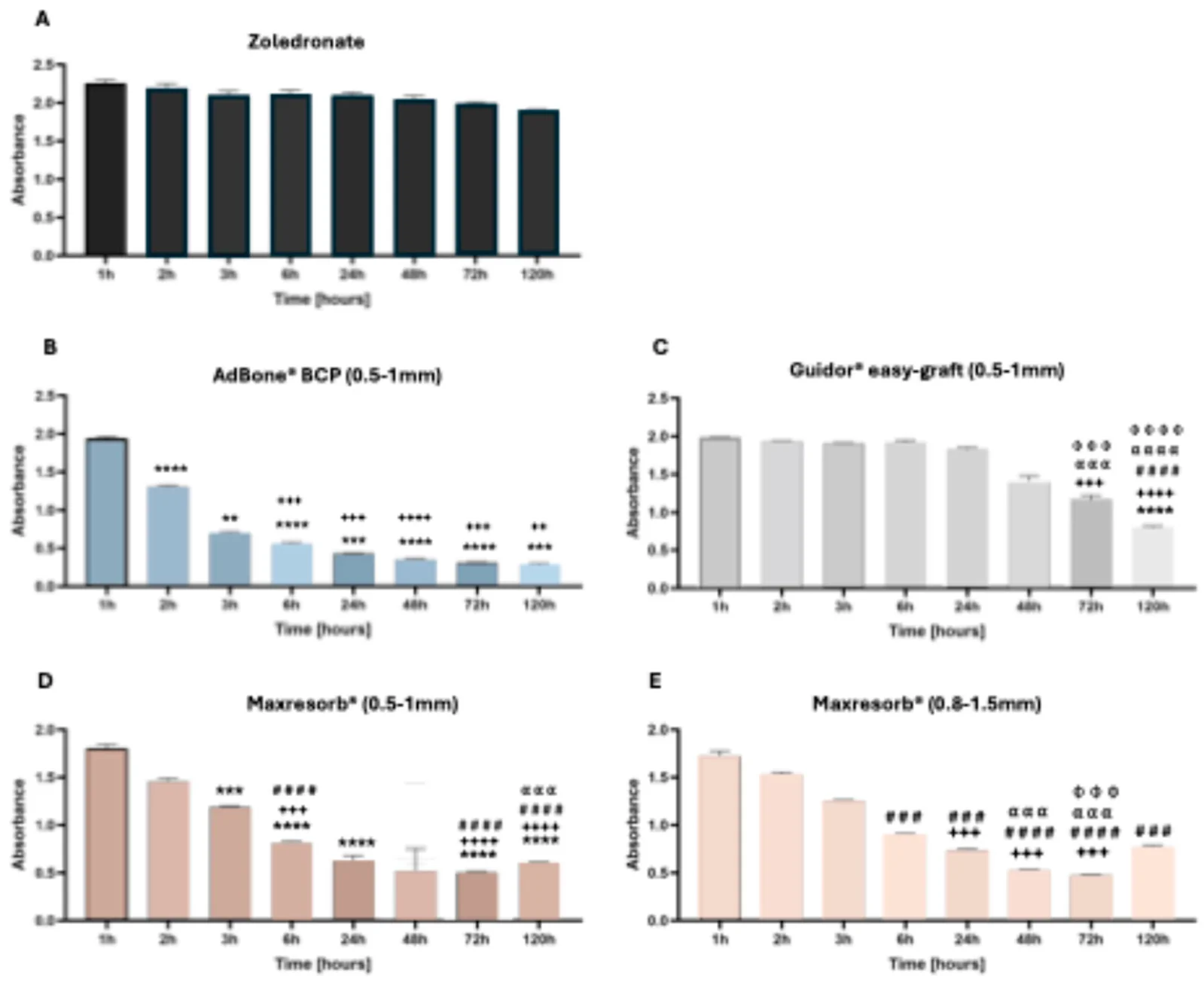
Columns represent mean absorbance values (210 nm) ± SD of (**A**) zoledronic acid (0.5 mM) control solution and of 0.5 mM zoledronic acid with 0.06 g of (**B**) AdBone^®^ BCP (0.5–1 mm), (**C**) Guidor^®^ easy-graft (0.5–1 mm), (**D**) Maxresorb^®^ (0.5–1 mm), and (**E**) Maxresorb^®^ (0.8–1.5 mm) over the 120 h evaluation period. Statistical significance refers to comparisons between each time point and specific earlier reference time points, denoted by distinct symbols when compared to 1 h (*), 2 h (+), 3 h (#), 6 h (α), and 24 h (ϕ); the number of repeated symbols indicates the significance level (three symbol: *p* < 0.001; four symbol: *p* < 0.0001).

Changes in the UV-Vis absorption spectrum over time indicate whether zoledronic acid was progressively adsorbed onto each ceramic material, with each material’s temporal profile compared against its own previous hours absorbance values, as represented in Figure 5. This within-material analysis allows the rate and progression of zoledronate uptake to be characterized independently for each formulation, prior to the between-material comparisons detailed below.

As shown in Figure 5A, the absorbance of the ZOL control solution remained stable throughout the 120 h period. In contrast, all four calcium phosphate materials produced a time-dependent decline in ZOL absorbance, though with markedly different onset, rate, and magnitude. AdBone^®^ BCP (Figure 5B) showed the earliest and most pronounced decline, reaching statistical significance relative to the 1 h baseline as early as 2 h, with absorbance continuing to decrease progressively through 120 h without any subsequent rebound. Maxresorb^®^ (0.5–1 mm; Figure 5D) also declined rapidly, reaching significance relative to baseline by 3 h, followed by a comparatively stable phase between 24 and 72 h. Maxresorb^®^ (0.8–1.5 mm; Figure 5E) followed a similar but slightly more gradual trajectory, with significance relative to baseline emerging from 6 h onward. Guidor^®^ easy-graft (Figure 5C) displayed a markedly different pattern, remaining statistically indistinguishable from its own 1 h baseline until 48 h, after which a significant decline finally emerged at 72 and 120 h, reflecting its delayed onset of zoledronate uptake. These within-material temporal trends, together with the between-material comparisons detailed below, are consistent with the distinct adsorption kinetics and mechanisms proposed for each formulation.

### 2.3. Kinetic Models

To further characterize the adsorption mechanism, the pseudo-first-order (PFO), pseudo-second-order (PSO), Elovich, and intraparticle diffusion (Weber–Morris) kinetic models were fitted to the adsorption data reported in Table 1, using their linearized forms. For Maxresorb^®^ formulations, fitting was restricted to the 1–72 h interval prior to the onset of partial re-release, as classical adsorption kinetic models do not account for subsequent desorption. Kinetic parameters and coefficients of determination (R^2^) are summarized in Table 2. AdBone^®^ BCP and both Maxresorb^®^ granulometries were best described by the pseudo-second-order model, yielding coefficients of determination of 0.999 (AdBone^®^ BCP), 1.000 (Maxresorb^®^ 0.5 mm), and 0.999 (Maxresorb^®^ 0.8 mm), with corresponding equilibrium adsorption capacities (qe) of 19.72, 17.55, and 17.84 mg/g, respectively. In contrast, Guidor^®^ easy-graft showed a comparatively poor fit to the pseudo-second-order model (R^2^ = 0.672) and was instead best described by the intraparticle diffusion model (R^2^ = 0.902), with a qe of 14.31 mg/g estimated by the pseudo-first-order model.

### 2.4. Elemental Analysis

Elemental analysis was carried out on a BCP pellet (0.06 g) after immersion in a zoledronate aqueous solution (0.5 mM, 120 h), with results presented in Table 3.

The nitrogen content in the pellets was 0.215%, 0.127% and 0.209% (*w*/*w*) for AdBone^®^ BCP, Guidor^®^ easy-graft and MaxResorb^®^, respectively (n = 6). Based on the nitrogen mass fraction of zoledronic acid (28.014 g/mol in 272.09 g/mol, representing 10.30% *w*/*w*), the estimated mass of adsorbed ZOL was 0.129 mg, 0.076 mg and 0.125 mg for AdBone^®^ BCP, Guidor^®^ easy-graft and MaxResorb^®^ 0.5 mm, respectively. Considering that the immersion solution contained 1.45 mg of zoledronic acid per 10 mL, analysis indicates that 85%, 50% and 83% of zoledronic acid was adsorbed by AdBone^®^ BCP, Guidor^®^ easy-graft and MaxResorb^®^ 0.5 mm, respectively, in close agreement with the UV/visible spectrophotometry results at 120 h. The quantification of adsorbed zoledronate was based specifically on nitrogen content, since neither hydroxyapatite nor β-tricalcium phosphate nor the PLGA polymer used in the Guidor^®^ easy-graft coating contains nitrogen in its chemical composition.

Carbon content was markedly higher in Guidor^®^ easy-graft (5.648% *w*/*w*) compared to AdBone^®^ BCP (0.608%) and MaxResorb^®^ 0.5 mm (1.104%), consistent with the presence of the PLGA polymer coating in this material. Hydrogen was detectable in AdBone^®^ BCP (0.297%) and Guidor^®^ easy-graft (0.164%) but below the detection limit (<100 ppm) in MaxResorb^®^ 0.5 mm. Sulfur was below the detection limit (<100 ppm) in all materials tested.

Elemental analysis of the drug-free calcium phosphate materials prior to zoledronate exposure was not performed because the nitrogen-based quantification of adsorbed zoledronate is unlikely to be confounded by this omission, since none of the tested materials or coating components (PLGA) contain nitrogen in their composition.

### 2.5. SEM-EDX

**Figure 6:**
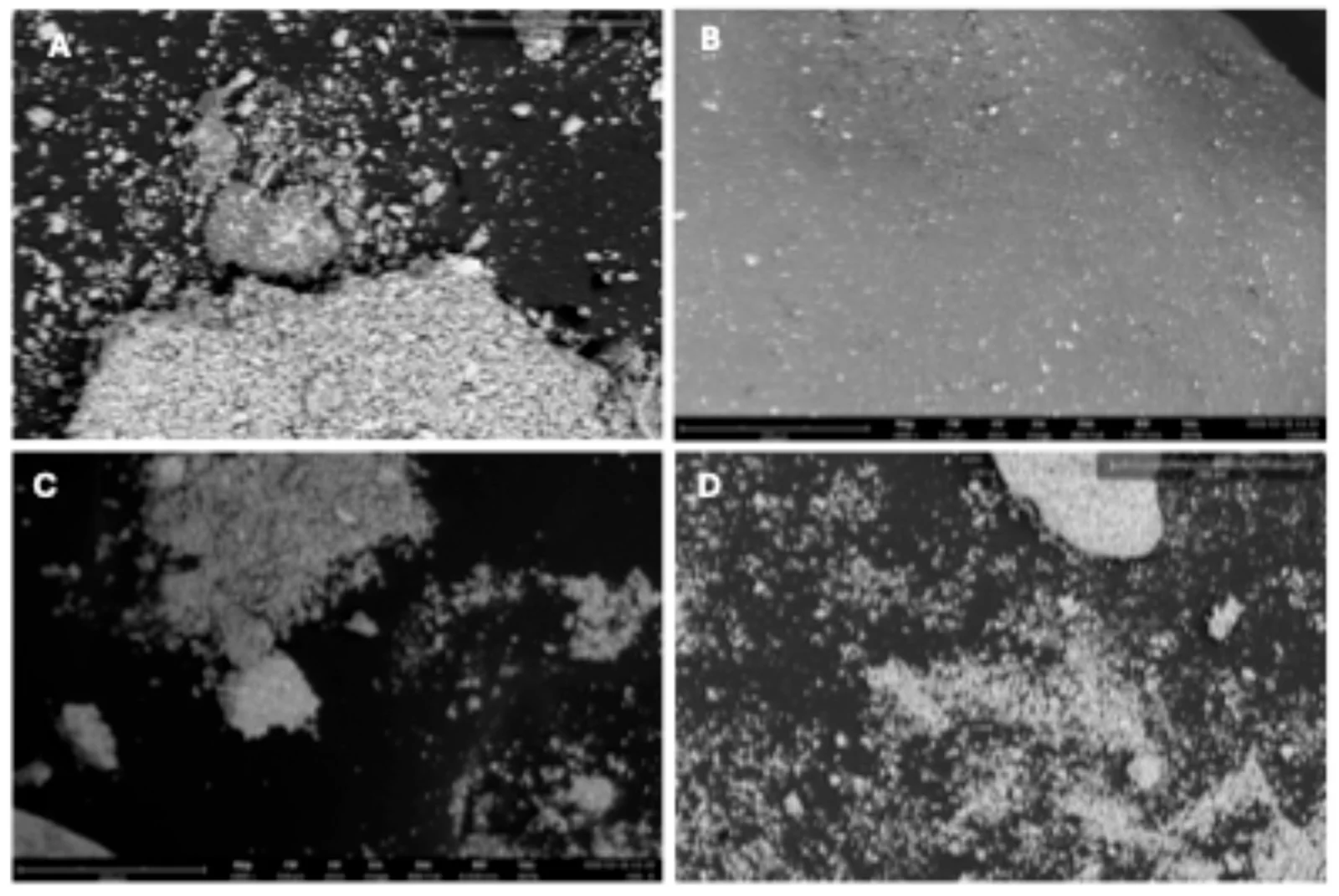
SEM images of (**A**) AdBone^®^BCP, (**B**) Guidor^®^ easy-graft (0.5–1 mm), (**C**) Maxresorb^®^ (0.5–1 mm) and (**D**) Maxresorb^®^ (0.8–1.5 mm) bone substitute granules, acquired at 1000× magnification (scale bar = 150 μm, as indicated in each image).

Figure 6 shows representative images of the surface morphology of the four materials investigated in this study after ZOL adsorption. Across all four materials, nitrogen was not detected by SEM-EDX at the surface locations examined, regardless of the surface Ca and P content.

The absence of a detectable nitrogen signal does not indicate the absence of nitrogen-containing compounds at the analyzed surfaces, since nitrogen not detected by SEM-EDX is a recognized limitation of EDX for light-element quantification (Z < 11) and is pronounced for nitrogen. The N Kα line (~0.392 keV) lies near the C Kα (~0.277 keV) and O Kα (~0.525 keV) lines, and the limited energy resolution of EDX detectors at these energies results in spectral overlap that masks the nitrogen signal [33]. Additionally, nitrogen exhibits a low X-ray fluorescence yield, with most excited atoms relaxing via Auger electron emission rather than characteristic X-ray emission, reducing detection of this element [34].

Nevertheless, the proposed diffusion-barrier hypothesis of Guidor^®^ easy-graft was supported by the EDX surface analysis. While Guidor^®^ easy-graft and Maxresorb^®^ have the same nominal composition, a divergent profile was observed. Maxresorb^®^ exhibited robust surface signals of both calcium (23.09 ± 2.04 wt%) and phosphorus (15.35 ± 0.58 wt%, n = 5). In contrast, Guidor^®^ easy-graft showed minimal surface calcium (0.66 ± 0.60 wt%) and phosphorus (0.29 ± 0.50 wt%, n = 5), with several individual measurements at or near the limit of detection. These results are consistent with the coated surface of Guidor^®^ easy-graft limiting the exposure of underlying reactive calcium phosphate to the EDX probe, in agreement with the intraparticle diffusion-limited kinetics observed for this material. EDX data relative to these materials are presented in Table 4. Moreover, the smooth, homogeneous surface observed in Figure 6B is consistent with the poly(lactide-co-glycolide) coating that surrounds the β-tricalcium phosphate granules in Guidor^®^ easy-graft.

## 3. Discussion

The present study evaluates the capacity of four commercially available biphasic calcium phosphate bone substitutes to sequester zoledronate, a nitrogen-containing bisphosphonate whose local cytotoxicity following dentoalveolar procedures is increasingly recognized as central to MRONJ pathogenesis [11,12,13,14].

Zoledronate adsorption onto calcium phosphate surfaces results from the convergence of complementary physicochemical interactions rather than from a single binding mode. The primary driving force is calcium ion chelation conferred by the two phosphonate groups of ZOL attached to the central carbon atom, which coordinate surface Ca^2+^ ions, while the hydroxyl group (-OH) present at the R1 position of the same carbon atom provides a third coordinating oxygen, together enabling a tridentate binding geometry [35]. This tridentate coordination forms stable five- and six-membered chelate rings with surface Ca^2+^, conferring markedly higher thermodynamic stability and mineral-binding affinity to zoledronate compared with bidentate-binding bisphosphonates [17,18,19,20].

Adsorption is governed by tridentate chelation and ion-exchange mechanisms, with the imidazole ring contributing secondary stabilization of the ZOL–ceramic complex [36,37,38,39]. The thermodynamic favorability of this exchange is attributable to the higher charge density and chelating geometry of the bisphosphonate group relative to inorganic phosphate. Additionally, the imidazole ring at R2 contributes to the secondary binding interaction with hydroxyl groups on the hydroxyapatite surface, further stabilizing adsorption [21]. These mechanisms effectively sequester zoledronate within the ceramic matrix, removing it from the bioavailable aqueous phase.

This distinction is mechanistically important because it separates two adsorption regimes with markedly different stability. True chemisorption, arising from phosphate substitution and tridentate chelation, is essentially irreversible under the conditions tested and accounts for the sustained, plateaued retention observed with AdBone^®^ BCP. Weaker, reversible physisorption, by contrast, is driven predominantly by electrostatic or van der Waals forces, without stable chelate or lattice incorporation, and is therefore more susceptible to thermodynamic re-equilibration and desorption over time. This latter regime offers a plausible explanation for the initial rapid capture of zoledronate, followed by its partial re-release over time, observed with Maxresorb^®^, which has a higher β-TCP proportion (40% β-TCP/60% HA) than AdBone^®^ BCP (25% β-TCP/75% HA).

In the context of zoledronate sequestration, materials that degrade too rapidly, such as pure β-TCP, risk releasing captured zoledronate back into the wound environment before soft tissue healing is complete. The hydroxyapatite (HA) component of BCP acts as a stable anchor, maintaining zoledronate sequestered within the socket throughout the critical healing window, while the β-TCP phase contributes the surface reactivity necessary for rapid initial adsorption [22,40,41].

Also for bone regeneration, biphasic calcium phosphate (BCP) ceramics are generally preferred over pure β-tricalcium phosphate (β-TCP) due to their superior control over resorption kinetics and structural stability. While β-TCP is highly biocompatible and osteoconductive, its rapid dissolution rate leads to premature loss of scaffold volume before sufficient new bone has formed. BCP addresses this limitation by combining the higher solubility of β-TCP, which promotes ion release and osteogenic stimulation, with the relative stability of hydroxyapatite (HA), which preserves scaffold integrity over a longer period. By adjusting the HA/TCP ratio, the degradation rate of BCP can be tailored to match the patient’s bone remodeling pace, ensuring a more predictable regenerative outcome [20,42,43].

The adsorption kinetics observed in the present study acquire direct clinical relevance when mapped onto the well-characterized biology of alveolar wound healing. In the first 24 h post-extraction, the socket is occupied by a fibrin-rich blood clot containing inflammatory cells and growth factors, with neutrophil migration facilitated by hyperemic periodontal ligament vessels. By 48–72 h, granulation tissue begins to replace the fibrin mesh, and the cells most critical for soft tissue repair (fibroblasts, endothelial cells and mesenchymal progenitors) are at their most active and simultaneously most vulnerable to the cytotoxic effects of free zoledronate. By day 5 (120 h), fibroblast proliferation from the periodontal ligament advances centripetally into the clot, with epithelial coverage established by day 7 and early osteoid trabeculae visible by day 10 [44].

At 24 h, when clot integrity and early cellular recruitment are paramount, AdBone^®^ BCP and Maxresorb^®^ had already reduced the free ZOL concentration by 80% and 70%, respectively, meaning that three-quarters or more of the potentially cytotoxic drug was captured and unavailable to soft tissue cells. Between 48 and 72 h, coinciding with the period of greatest fibroblast and endothelial cell vulnerability, both materials maintained reductions of 75–84%, providing a substantial pharmacological buffer throughout this critical window. At 120 h, AdBone^®^ BCP sustained 85% adsorption, while Maxresorb^®^ declined to 60–70%, suggesting partial re-release; however, given that fibroblast proliferation at this stage is already advancing away from the bone surface toward the socket periphery, this modest reduction in retention capacity is unlikely to critically compromise healing.

The 120 h time point already exceeds the 48–72 h window identified as the period of greatest cellular vulnerability to zoledronate cytotoxicity; by this stage, granulation tissue coverage is already well established, and fibroblast proliferation is advancing toward the socket periphery, meaning that the partial re-release observed for Maxresorb^®^ occurs largely outside the critical window and is therefore unlikely to pose a significant cytotoxic risk. Guidor^®^ easy-graft, by contrast, achieved only a 12–13% reduction through the first 24 h and 32–41% at 48–72 h, precisely the window of greatest cellular vulnerability, rendering it ineffective as a zoledronate buffer during the most critical phase of alveolar repair.

This temporal alignment between adsorption efficacy and wound healing biology is consistent with our previous in vitro and in vivo findings, in which free ZOL significantly impaired gingival fibroblast viability, migration and cell cycle progression, while the presence of BCP restored outcomes to levels comparable to control groups [15], and enable cicatrisation of extraction wounds in an Biphosphonates related osteonecrosis of the jaw animal model [25,45]. By capturing zoledronate as it is released from alveolar bone into the acidic, inflammatory wound environment [38,46], calcium phosphate bone substitutes act as a local pharmacological buffer, reducing bioavailable drug concentration during the window of greatest cellular vulnerability and thereby creating conditions more favorable to normal socket closure and tissue repair [47].

Among the materials tested, AdBone^®^ BCP (75% HA/25% β-TCP) demonstrated the most favorable adsorption profile. Free ZOL in solution decreased by 14% at 1 h, 40% at 2 h and 67% at 3 h, continuing to fall to 74% at 6 h, 80% at 24 h and stabilizing at 83–85% from 48 h through to 120 h, with no evidence of re-release. This progressive, monotonic decrease is consistent with stable chemisorption driven by the biphasic composition of the material: the β-TCP component, with its higher solubility and faster dissolution rate, provides a high density of reactive surface sites for rapid initial phosphonate exchange, while the hydroxyapatite phase provides durable anchoring over time [20,42,43]. The plateau sustained at 85% through 120 h covers the entire biologically critical window of alveolar healing, encompassing active fibroblast proliferation, epithelial migration and early osteoid formation, making AdBone^®^ BCP the most suitable candidate among the tested materials for clinical use in extraction sockets in patients under bisphosphonate therapy.

Maxresorb^®^, tested at two granulometries (0.5–1.0 mm and 0.8–1.5 mm), demonstrated effective zoledronate adsorption broadly comparable to AdBone^®^ BCP, but with two notable distinctions. Both granulometries exhibited slightly faster initial uptake at 1 h (20% and 24% vs. 14%), followed by slower progression through 3 h (43% and 40% vs. 67%), converging to closer values between 24 and 72 h (70–75% vs. 80–85%). Unlike AdBone^®^ BCP, however, both granulometries showed a decline in retention at 120 h, to 70% for 0.5–1.0 mm and 60% for 0.8–1.5 mm, indicating partial re-release, more pronounced with the larger particle size.

No substantial difference in adsorption kinetics was observed between the two granulometries of Maxresorb^®^ through 72 h, consistent with the initial, rapid chemisorption phase being governed primarily byinternal microporosity rather than by macroscopic granule size [20,41,43,48]. The greater re-release with the larger granulometry at 120 h (17%, vs. 7% for the smaller granulometry) suggests its lower external surface-area-to-volume ratio, a geometric consequence of increased particle radius, which could reduce the relative density of stable long-term chemisorption sites, accessible at the granule surface, favoring a proportionally greater contribution of weaker, reversible physical interactions susceptible to re-equilibration over time [20,42,45,49]. However, as no direct specific surface area measurements (e.g., BET analysis) were performed for the two granulometries in this study, this explanation remains speculative [19,20,40,41,43,48,50].

The partial re-release observed with MaxResorb^®^ at 120 h suggests that a fraction of the adsorbed drug was retained through weaker, reversible physical interactions such as electrostatic forces or Van der Waals interactions, rather than stable chemisorption, with thermodynamic re-equilibration favoring its return to the aqueous phase over time, possibly related to its higher proportion of β-TCP (40%) [51]. The sustained 85% adsorption achieved by AdBone^®^ BCP, by contrast, is consistent with predominantly irreversible chemisorption at a higher density of reactive surface sites, possibly related to its higher proportion of HA (75%), conferring a more durable pharmacological buffering effect within the surgical wound environment. Additionally, the lack of evidence of re-release is also consistent with the literature indicating that bisphosphonates adsorbed onto apatitic calcium phosphate surfaces via chemisorption are, in the majority, irreversibly bound and not spontaneously released by dilution or simple washing [45].

The distinct retention stability observed between AdBone^®^ BCP and Maxresorb^®^ may be explained not only by their differing dissolution kinetics, but also by a compositionally driven difference in the intrinsic stability of the zoledronate–calcium phosphate complex itself. Beyond providing a greater density of readily accessible Ca^2+^ sites, the higher HA content of AdBone^®^ BCP (75% HA/25% β-TCP) may confer an additional layer of binding stabilization not equally available to the more β-TCP-rich Maxresorb^®^ (60% HA/40% β-TCP). Computational studies have attributed part of the strong affinity of bisphosphonates for hydroxyapatite to hydrogen bonding between the bisphosphonate’s own R1 hydroxyl group and structural hydroxyl/oxygen groups present within the hydroxyapatite lattice [49]. Because β-TCP (Ca_3_(PO_4_)_2_) lacks these structural hydroxyl groups, zoledronate binding to β-TCP-rich surfaces may rely more heavily on direct Ca^2+^ chelation alone, without the additional hydrogen-bonding network available at HA-rich surfaces. This could render the adsorbed zoledronate/β-TCP complex intrinsically more susceptible to displacement, compounding the effect of β-TCP’s greater solubility discussed above: as the more soluble β-TCP fraction of Maxresorb^®^ continues to dissolve over time, the resulting release of structural Ca^2+^ and PO_4_^3−^ ions may further destabilize these comparatively weaker complexes, offering a plausible, literature-supported explanation for the partial re-release observed for Maxresorb^®^ at 120 h, in contrast with the stable, essentially irreversible retention sustained by the HA-dominant AdBone^®^ BCP throughout the same period.

A related, though mechanistically distinct, structure–function relationship may account for the markedly delayed uptake observed for Guidor^®^ easy-graft. Rather than reflecting differences in the underlying HA/β-TCP composition, this delay is attributed to its PLGA-based surface coating, which physically restricts zoledronate access to the reactive calcium phosphate surface beneath, consistent with the diffusion-limited kinetics identified above (intraparticle diffusion model, R^2^ = 0.901) and with the near-absent surface Ca and P signals detected by EDX. While both Maxresorb^®^ and Guidor^®^ exhibited reduced zoledronate retention relative to AdBone^®^ BCP, the underlying mechanisms differ: for Maxresorb^®^, this reflects an intrinsically less stable, more readily reversible binding process linked to its higher β-TCP content; for Guidor^®^, it instead reflects a physical barrier delaying, rather than preventing, access to an otherwise reactive calcium phosphate surface.

Guidor^®^ easy-graft consists of calcium phosphate granules coated with a resorbable PLGA polymer layer containing acetyl tributyl citrate (ATBC) as a plasticizer. In clinical use, activation with a specific liquid softens this coating, exposing the underlying calcium phosphate surface. In the present study, Guidor^®^ easy-graft was tested with prior activation, meaning the PLGA coating was softened.

The absorbance of the ZOL/Guidor solution remained within 10–13% of the ZOL control through 24 h, reaching only 32%, 41% and 58% reduction at 48, 72 and 120 h, respectively. The negligible adsorption at early time points is consistent with the polymer coating eventually acting as a physical barrier, preventing zoledronate from accessing the ion-exchange sites of the underlying calcium phosphate surface. The gradual reduction observed from 48 h onwards likely reflects slow hydrolytic degradation of the PLGA layer upon prolonged aqueous exposure, as Middleton & Tipton (2000) demonstrate, rather than true chemisorption [51]. Whether activation prior to contact with zoledronate could restore adsorption kinetics comparable to uncoated ceramics remains an open and clinically relevant question warranting future investigation, particularly given the use of similar coated materials in socket preservation procedures in patients under bisphosphonate therapy. The absence of a detectable nitrogen signal by SEM-EDX, despite the successful nitrogen-based quantification of adsorbed zoledronate by elemental analysis, does not necessarily indicate the absence of nitrogen at the analyzed surfaces. Rather, it reflects a well-recognized instrumental limitation of the technique EDX in the detection of light elements (Z < 11), particularly nitrogen. The N Kα line (~0.392 keV) lies in close proximity to the C Kα (~0.277 keV) and O Kα (~0.525 keV) lines, and the limited energy resolution of EDX detectors in this energy range frequently results in peak overlap that obscures the nitrogen signal [33]. Furthermore, nitrogen exhibits an intrinsically low X-ray fluorescence yield, with most excited atoms relaxing via Auger electron emission rather than characteristic X-ray emission, further limiting detection sensitivity for this element by EDX [34].

In contrast, elemental analysis by combustion and thermoconductivity detection is not subject to these limitations and remains the more reliable method for nitrogen quantification, as reflected by the close agreement between the CHNS-based adsorption estimates and the UV/visible spectrophotometry results reported above. To directly examine the surface-level basis for this diffusion-limited behavior, SEM-EDX analysis was performed on Guidor^®^ easy-graft and Maxresorb^®^, the two materials with the most divergent kinetic and re-release profiles. Maxresorb^®^ displayed robust surface signals of both calcium (23.09 ± 2.04 wt%) and phosphorus (15.35 ± 0.58 wt%), consistent with an exposed, uncoated calcium phosphate surface and yielding a Ca/P ratio (1.50) compatible with a β-TCP-rich composition. In marked contrast, Guidor^®^ easy-graft showed minimal surface calcium (0.66 ± 0.60 wt%) and phosphorus (0.29 ± 0.50 wt%) with a near-absence of a detectable calcium phosphate signal at its surface. This provides direct physical evidence that its PLGA-based coating substantially overshadows the underlying reactive ceramic, corroborating the diffusion-limited kinetics inferred from the intraparticle diffusion model fit above and offering a more direct, mechanistic explanation for its delayed and reduced zoledronate uptake, particularly during the critical early healing window. Quantitative kinetic modeling supports the mechanistic interpretation proposed above. AdBone^®^ BCP and both Maxresorb^®^ granulometries were best fitted by the pseudo-second-order model (R^2^ ≥ 0.999), indicating that the rate-limiting step of zoledronate uptake is governed by chemisorption, consistent with the tridentate chelation and ion-exchange mechanisms described earlier. In contrast, Guidor^®^ easy-graft showed a poor fit to the pseudo-second-order model (R^2^ = 0.672) and was instead better described by the intraparticle diffusion model (R^2^ = 0.902), indicating that its adsorption is limited by diffusional access rather than by the intrinsic reactivity of the calcium phosphate surface. This finding provides quantitative support for our proposal that the PLGA-based coating of Guidor^®^ easy-graft constitutes a physical barrier restricting zoledronate diffusion to the underlying reactive sites, delaying rather than preventing chemisorption, consistent with its markedly slower kinetics through 24 h followed by later, gradual uptake.

The present physicochemical findings should be interpreted within the broader body of evidence supporting this therapeutic strategy. In vitro studies from our group demonstrated that BCP protects gingival fibroblasts from ZOL-induced cytotoxicity across multiple endpoints including cell viability, migration, cell cycle progression and apoptosis, corroborated by Bullock et al. (2020) [15,16]. Subsequent in vivo validation in a Wistar rat extraction model confirmed that placement of calcium phosphate ceramics in the alveolus reduced ZOL toxicity and promoted histologically confirmed healing [16], with independent corroboration provided by Hadad et al. (2022) [27]. The present study extends these findings by demonstrating that effective zoledronate sequestration is not restricted to AdBone^®^ BCP but is a property shared by other biphasic calcium phosphate substitutes, broadening the clinical applicability of this approach regardless of which specific commercial product is available to the clinician. Further in vitro and in vivo studies are warranted to validate the clinical efficacy of these materials in preventing bisphosphonate-related osteonecrosis following dentoalveolar procedures.

The spectrophotometric findings were independently confirmed by elemental analysis of the BCP pellet after immersion in a 0.5 mM ZOL solution for 120 h. Nitrogen quantification yielded an estimated ZOL uptake of 0.129 mg, 0.076 mg and 0.125 mg for AdBone^®^ BCP, Guidor^®^ easy-graft and Maxresorb^®^ 0.5 mm, respectively, corresponding to 85%, 50% and 83% of the initial zoledronic acid amount. The results were closely aligned with the reduction observed by UV/visible spectrophotometry at 120 h (85%, 57% and 70%, respectively), with the exception of Maxresorb^®^ 0.5 mm, for which the elemental analysis yielded a higher estimate (83% vs. 70%). This discrepancy may reflect the dynamic nature of the adsorption–desorption equilibrium: while spectrophotometry measures free ZOL concentration in solution at each individual time point, elemental analysis quantifies the total nitrogen retained in the pellet at the end of the experiment. For Maxresorb^®^, which exhibited partial re-release between 72 and 120 h in the spectrophotometric data, the elemental analysis may therefore capture a higher bound fraction than the final spectrophotometric time point alone. Nonetheless, both methods consistently confirm effective zoledronate sequestration by AdBone^®^ BCP and Maxresorb^®^ and negligible retention by Guidor^®^ easy-graft relative to the other two materials. This dual-method confirmation reinforces the reliability of the quantification approach. These values are comparable to those reported by Josse et al. (2005) [52], who used ^31^P NMR to quantify ZOL adsorption by BCP (75HA/25TCP) and found the nitrogen (0.29%) and carbon (0.64%) percentages in close agreement with our values for AdBone^®^ BCP (0.21% N and 0.608% C).

As an in vitro investigation, the experimental conditions employed, including fixed zoledronate concentration, standardized ceramic mass, constant pH and controlled temperature of 25 °C, do not fully replicate the complexity of the in vivo extraction socket, where temperature dynamic pH fluctuations (between pH 5.5/6 in the presence of infection and pH 6.8 in a physiologic pos extraction state) [53,54] physiological temperature (37 °C), protein adsorption and inflammatory mediators may significantly influence adsorption kinetics and zoledronate retention. Although the zoledronate–calcium phosphate interaction is primarily governed by a strong, affinity-driven ion-exchange mechanism, expected to occur robustly across this temperature range, physiological temperature could accelerate the observed kinetics, potentially narrowing the time window required to reach the adsorption plateaus reported here [45].

Beyond the mechanistic hypotheses discussed above, an important direction for future research will be to directly correlate the adsorption efficiency reported here with the specific microstructural and compositional parameters of each commercial material, surface area measurements (BET analysis), pore size distribution and porosimetry, and crystallinity (e.g., by X-ray diffraction). Direct surface-analytical techniques, such as X-ray photoelectron spectroscopy (XPS) or Fourier-transform infrared spectroscopy (FTIR), would be required to directly confirm the proposed adsorption mechanism, and their use is recommended in future studies. Additionally, dynamic blood flow, protein adsorption and inflammatory mediators present in the in vivo wound environment are not replicated by the present static, single-protein-free model. Although previous works demonstrate the impact of this reaction in cellular models and in in vivo models, as was referred to previously, it remains an important direction for future work to confirm the translational relevance of the adsorption capacities reported here.

Nevertheless, based on the present findings, and in the in vitro and in vivo studies, immediate socket grafting with AdBone^®^ BCP at the time of tooth extraction may represent a promising, implementable preventive strategy for patients undergoing bisphosphonate therapy who require dental extraction, given its sustained zoledronate sequestration capacity throughout the critical 120 h healing window. From a surgical standpoint, the proposed protocol would involve atraumatic tooth extraction thorough debridement and irrigation of the socket, grafting with the calcium phosphate material to fill the extraction socket, and primary or partial soft tissue closure to promote graft stabilization and reduce the risk of exposure, in line with established socket preservation protocols already routinely used for ridge preservation in implant dentistry, and to which this application would represent a direct extension.

## 4. Materials and Methods

### 4.1. Zoledronate

Zoledronic acid (1-Hydroxy-2-(imidazol-1-yl) ethane-1,1-bisphosphonic acid, Hangzhou Pharm & Chem Co., Ltd., Hangzhou, China) was solubilized in ultrapure water to obtain a 0.5 mM solution. Further concentrations were obtained by serial dilution. The pH was measured using a model 3510 pH meter (Jenway, Stone, UK).

### 4.2. Spectrometric Quantification of Imidazole and Zoledronic Acid

Most bisphosphonates do not contain chromophore groups in their structure, which prevents their analysis by conventional spectrophotometric techniques. However, the molecule of zoledronic acid includes an imidazole ring in its structure, which allows its detection by spectrophotometry. Therefore, spectrometric quantification of an aqueous imidazole solution was performed to confirm that the absorbance was related to the imidazole ring present in zoledronate, using similar concentrations. Starting from an initial imidazole solution at a concentration of 1 mM, dilutions with distilled water were performed to achieve the following concentrations: 0.5 mM, 0.3 mM, 0.1 mM, and 0.05 mM. The experimental data allowed for a linear fit using Origin 8.0 software, comparing it with that of zoledronic acid at the same concentrations, between 190 and 350 nm. Starting from the initial zoledronic acid solution at a concentration of 0.5 mM, dilutions with distilled water were prepared to achieve concentrations of: 0.5 mM, 0.3 mM, 0.1 mM, and 0.05 mM. The experimental data were analyzed with a linear fit using Origin 8.0 software, resulting in the standard curve for zoledronic acid. The stability of the samples was also assessed by evaluating their visual appearance and absorbance at 24, 48, 72, 96, and 120 h, both in the presence and absence of light.

### 4.3. Calcium Phosphate Ceramics

Calcium phosphate ceramics, composed of hydroxyapatite (HA) and β-TCP, from four commercial brands were used: 75% hydroxyapatite (HA) and 25% β-TCP AdBone^®^ BCP (Medbone^®^, Medical Devices, Lisbon, Portugal, Ref.BCP010502G, Lot 00211); 60% hydroxyapatite (HA) and 40% β-TCP Guidor easy-graft Crystal (Sunstar Ref. C15-102; Lot 21-062–activated with the activator liquid); 60% hydroxyapatite (HA) and 40% β-TCP Maxresorb^®^ 0.5–1.0 mm (Klockner Ref.20005; Lot 2432-1) and 60% hydroxyapatite (HA) and 40% β-TCP Maxresorb^®^ 0.8–1.5 mm (Klockner Ref. 20105; Lot 2337-4). The four biomaterials are currently used in dentistry as bone substitutes, are of 100% synthetic origin, and have a manufacturer-declared porosity of 60%, a pore size of 300–500 microns and a mechanical strength of 3.0 MPa.

### 4.4. Adsorption of Zoledronic Acid to Calcium Phosphate Ceramics

All adsorption experiments were carried out at a controlled room temperature of 25 °C. Samples of 0.06 g of each biomaterial were placed in tubes with ten milliliters of the zoledronic acid solution (0.5 mM). Tubes were kept under magnetic stirring (120 rpm) for 1 h, 2 h, 3 h, 6 h, 24 h, 48 h, 72 h and 120 h of reaction. After measurement, the samples were returned to their original solution to avoid altering the concentration. The experiments were performed 3 times in duplicate.

### 4.5. UV/Visible Spectroscopy

The absorption spectrum of each solution was recorded before the addition of the ceramics and after the corresponding conditioning period. All supernatant samples were centrifuged prior to spectrophotometric analysis (8000 rpm, 2 min).

The absorption spectra in the UV/visible region were recorded using a Hitachi Model U 2000 spectrophotometer (Hitachi, Tokyo, Japan). UV/visible absorption spectroscopy is often used for the quantitative determination of compounds containing absorbing groups, with Beer–Lambert’s law providing the mathematical basis for determining concentration by measuring the intensity of light absorbed by the sample between 190 and 350 nm.

### 4.6. Kinetic Models

To further characterize the adsorption mechanism, the amount of zoledronate adsorbed per gram of ceramic at each time point (qt, mg/g) was calculated from the percentage of absorbance decrease reported in Table 1, based on the initial zoledronate concentration (0.5 mM), the solution volume (10 mL), the ceramic mass (0.06 g), and the molecular weight of zoledronic acid (272.09 g/mol). The pseudo-first-order (PFO), pseudo-second-order (PSO), Elovich, and intraparticle diffusion (Weber–Morris) kinetic models were fitted to the resulting qt-versus-time data using their linearized forms:

Pseudo-first-order: ln(qe − qt) = ln(qe) − k_1_t

Pseudo-second-order: t/qt = 1/(k_2_qe^2^) + t/qe

Elovich: qt = (1/β)ln(αβ) + (1/β)ln(t)

Intraparticle diffusion (Weber–Morris): qt = kidt^0.5^ + C

where qe and qt (mg/g) represent the amounts of zoledronate adsorbed at equilibrium and at time t, respectively; k_1_ (h^−1^) and k_2_ (g·mg^−1^·h^−1^) are the pseudo-first- and pseudo-second-order rate constants; α (mg·g^−1^·h^−1^) and β (g/mg) are the initial adsorption rate and desorption constants of the Elovich model; and kid (mg·g^−1^·h^−0^·^5^) and C (mg/g) are the intraparticle diffusion rate constant and the intercept related to boundary layer thickness, respectively. For each material, the experimental qt value at the final time point considered was used as the estimate of qe for the pseudo-first-order model. For Maxresorb^®^ formulations, model fitting was restricted to the 1–72 h interval, prior to the onset of partial re-release, since classical adsorption kinetic models do not account for a subsequent desorption phase. Linear regression parameters and coefficients of determination (R^2^) for each model and material were obtained using GraphPad Prism 10.4 and are summarized in Table 2.

### 4.7. Elemental Analysis

Microanalysis was used to determine the constituent elements of a particular molecule and their proportions, determining the empirical formula of the organic compounds. For this analysis, a Fisons O.CHS apparatus (Fisons Instruments, Milan, Italy) was used to analyze the pellet resulting from the adsorption reaction of zoledronic acid to calcium phosphate ceramics after maintaining a zoledronic acid solution at a concentration of 0.5 mM in a rotary apparatus with 0.06 g of BCP for 120 h. The pellet was subsequently dried in an oven at 50 °C.

### 4.8. SEM-EDX

Surface elemental composition was assessed by scanning electron microscopy coupled with energy-dispersive X-ray spectroscopy (SEM-EDX) for all four commercially available biphasic calcium phosphate materials evaluated in this study after zoledronate adsorption (AdBone^®^ BCP, Guidor^®^ easy-graft, Maxresorb^®^ 0.5–1 mm, and Maxresorb^®^ 0.8–1.5 mm) using a Thermo Scientific Phenom XL (Thermo Fisher Scientific, Eindhoven, Netherlands) equipped with an EDX detector. Samples were mounted on aluminum stubs using carbon adhesive tape and analyzed without conductive coating. Imaging and elemental analysis were performed at an accelerating voltage of 10 kV. For each material, elemental composition, including calcium, phosphorus, carbon, oxygen and nitrogen, was quantified at five representative surface locations, and calcium and phosphorus content was expressed as weight percentage (wt%), mean ± standard deviation. Data acquisition and quantification were performed using the software of the equipment.

### 4.9. Statistical Analysis

Statistical analysis was performed using GraphPad Prism 10.4 (GraphPad Software, LLC, San Diego, CA, USA). Data normality was assessed using the Shapiro–Wilk test, and homogeneity of variances was evaluated using the Brown–Forsythe test. Comparisons between biomaterials were performed independently. For normally distributed data with homogeneous variances, ordinary one-way analysis of variance (ANOVA) was conducted, followed by Tukey’s Honest Significant Difference (HSD) post hoc test for multiple comparisons.

When homogeneity of variances was not met, Welch’s ANOVA was applied, followed by Dunnett’s T3 multiple-comparisons test, as implemented in the software. For non-normally distributed data, the Kruskal–Wallis test was used, followed by Dunn’s multiple-comparisons test.

Time-course analyses within each biomaterial were performed using a mixed-effects model (REML), with repeated measurements matched within each independently prepared sample, which constituted the experimental unit throughout the adsorption assay. The Geisser–Greenhouse correction was applied to account for potential violations of sphericity. When significant effects were detected, Šídák’s multiple-comparisons test was used for pairwise comparisons between time points.

Statistical significance was established at a family-wise confidence level of 95% (*p* < 0.05), and all post hoc analyses incorporated appropriate corrections for multiple comparisons as described above.

## 5. Conclusions

The present study demonstrates that commercially available synthetic biphasic calcium phosphate bone substitutes are capable of adsorbing zoledronate through stable chemisorption, with kinetics well matched to the critical window of alveolar wound healing. AdBone^®^ BCP showed the most favorable profile, achieving rapid and stable sequestration of 85% of free zoledronate through 120 h without re-release. Maxresorb^®^ achieved clinically meaningful adsorption (70–75%) throughout most of the evaluation period, although partial re-release at 120 h warrants consideration. Guidor^®^ easy-graft showed the lowest adsorption during the critical early healing phase; this result reflects the role of the intact PLGA coating as a physical barrier to the underlying calcium phosphate surface, which, even after activation with the recommended liquid, was insufficient to restore adsorption kinetics comparable to uncoated materials. Spectrophotometric findings were independently confirmed by elemental analysis, with both methods yielding consistent estimates of zoledronate uptake. These findings support the use of biphasic calcium phosphate bone substitutes as a local pharmacological strategy to reduce free zoledronate concentration at the surgical wound site.

## Figures and Tables

**Table 1:** Percentages of absorbance decrease (% ± SD) in the presence of the biomaterials in a 0.5 mM zoledronate aqueous solution.

	1 h	2 h	3 h	6 h	24 h	48 h	72 h	120 h
AdBone^®^ BCP	13.79 ± 4.11	40.14 ± 3.88	66.89 ± 3.06	73.77 ± 2.32	79.27 ± 1.17	82.70 ± 1.51	84.16 ± 0.40	85.22 ± 2.10
Guidor^®^	12.15 ± 1.88	11.69 ± 3.47	9.00 ± 2.73	9.12 ± 2.23	12.62 ± 6.16	31.57 ± 10.45	41.43 ± 7.07	57.76 ± 2.27
Maxresorb^®^ 0.5	20. 04 ± 7.28	33.24 ± 5.13	43.16 ± 3.69	61.25 ± 2.28	69.77 ± 6.03	74.02 ± 16.49	74.76 ± 0.96	68.08 ± 0.72
Maxresorb^®^ 0.8	23.67 ± 6.10	30.14 ± 4.61	40.21 ± 3.85	57.42 ± 2.32	64.49 ± 2.09	74.07 ± 1.56	76.12 ± 0.79	59.22 ± 1.24

**Table 2:** Adsorption kinetic model parameters (pseudo-first-order, pseudo-second-order, Elovich, and intraparticle diffusion) fitted to the zoledronate adsorption data reported in Table 1, using their linearized forms.

Material	PFO k_1_ (h^−1^)	PFO qe (mg/g)	PFO R^2^	PSO k_2_ (g·mg^−1^·h^−1^)	PSO qe (mg/g)	PSO R^2^	Elovich R^2^	Intrapart. Diff. R^2^
AdBone^®^	0.0505	7.06	0.862	0.0210	19.72	0.999	0.742	0.531
Guidor^®^	0.0146	11.65	0.928	0.0022	14.31	0.672	0.702	0.902
Maxresorb^®^ 0.5 mm	0.0854	9.27	0.959	0.0237	17.55	1.000	0.914	0.751
Maxresorb^®^ 0.8 mm	0.0635	10.13	0.953	0.0184	17.84	0.999	0.955	0.843

**Table 3:** Mean of 6 experiments of the elemental analysis of BCP pellet after immersion in a zoledronate aqueous solution.

Sample	% N (*w*/*w*)	% C (*w*/*w*)	% H (*w*/*w*)	% S (*w*/*w*)
AdBone^®^ BCP	0.215	0.608	0.297	<100 ppm
Guidor^®^ easy-graft	0.127	5.648	0.164	<100 ppm
MaxResorb^®^ 0.5 mm	0.209	1.104	<100 ppm	<100 ppm

**Table 4:** Individual EDX measurement points (weight percentage, wt%) of calcium (Ca) and phosphorus (P) obtained for Guidor^®^ easy-graft and Maxresorb^®^ (0.5–1 mm), used to calculate the mean ± SD values reported in the main text.

Material	Ca					P				
Guidor^®^ easy-graft	0.25	1.69	0.5	0.25	0.6	0	0	0.3	0	1.16
Maxresorb^®^ (0.5–1 mm)	20.08	24.31	24.31	24.88	21.89	16.07	15.02	15.02	15.89	14.77

## Data Availability

The original contributions presented in this study are included in the article. Further inquiries can be directed to the corresponding authors.

## Abbreviations

The following abbreviations are used in this manuscript:

BP	Bisphosphonate
MRONJ	Medication-related osteonecrosis of the jaw
ZOL	Zoledronate
ADBONE	AdBone^®^ BCP (0.5–1 mm)
GUIDOR	Guidor^®^ easy-graft (0.5–1 mm)
MAXRES 0.5	Maxresorb^®^ (0.5–1 mm)
MAXRES 0.8	Maxresorb^®^ (0.8–1.5 mm)
PLGA	poly(lactide-co-glycolide)
